# Proteoglycans in Prostate Cancer Progression and Therapy Resistance

**DOI:** 10.3390/medicina61122112

**Published:** 2025-11-27

**Authors:** Ivana Samaržija

**Affiliations:** Laboratory for Epigenomics, Division of Molecular Medicine, Ruđer Bošković Institute, 10000 Zagreb, Croatia; ivana.samarzija@irb.hr

**Keywords:** proteoglycan, prostate cancer, cancer progression, therapy resistance, biomarker, extracellular matrix, tumor microenvironment

## Abstract

The extracellular matrix (ECM) is a complex noncellular network of (macro-)molecules that surrounds and supports diverse cells in tissues and organs. In cancer, ECM is a part of the tumor microenvironment (TME) that embeds its cellular components including cancer cells and the neighboring non-cancerous stromal cells such as fibroblasts, endothelial, and immune cells. Given the complexity of players and interactions that the ECM participates in and is exposed to in the TME, it does not come as a surprise that many of the processes that drive cancer progression take part precisely in the ECM compartment of the TME. Along with diverse glycoproteins and collagens, proteoglycans (PGs) are among the main components of the core ECM. PGs are composed of a protein core to which glycosaminoglycan chains are attached. Considering the structural diversity of these molecules and their ‘hybrid’ nature, it is not surprising that they are involved in a variety of processes that are vital for surrounding cells. Moreover, they are secreted by both cancer and stromal cells, contributing to the complexity of interactions in the TME. In prostate cancer, PGs have been shown to be involved in many steps of its progression; the most prominent examples include the seemingly tumor-promoting roles of versican, perlecan, and biglycan, and the tumor-suppressive roles of decorin and betaglycan. The role of syndecan 1 is a bit more complex; namely, the nature of its role is context dependent. In this narrative review article, the roles of PGs in prostate cancer progression and therapy resistance are discussed in more detail.

## 1. Introduction

The tumor microenvironment (TME) is a collective name for components found in tissue surrounding tumors; it is composed of cancer cells and the neighboring non-cancerous stromal cells such as fibroblasts, endothelial, and immune cells [1]. The cellular components of the TME are embedded in the extracellular matrix (ECM), a complex network of (macro-)molecules that surrounds and supports diverse cells in tissues and organs [2,3]. The increasing knowledge on the importance of the TME in cancer progression has led to recognition that most of the hallmarks of cancer are enabled and sustained by TME components [4,5]. Since ECM is a vital part of the TME, it does not come as a surprise that it is involved actively in many steps of tumor progression [6,7].

Along with many glycoproteins and collagens, proteoglycans (PGs) are among the main components of the core ECM. PGs show ‘hybrid’ nature, that is, they are composed of a protein core to which glycosaminoglycan chains are attached. Their diverse structure enables numerous interactions and participation in a multitude of processes which are, in particular, widely studied in reference to different steps of cancer progression (reviewed in [8,9,10,11]).

Prostate cancer (PC) is among the leading cancers in terms of incidence and mortality [12]. PGs have been studied in the context of PC in order to better understand its routes of progression and resistance to therapy and to suggest novel targets (reviewed in [13,14]). This review article summarizes the current knowledge on the role of PGs in PC progression and therapy resistance, with an emphasis on the most recent studies.

### Search Strategy and Selection Criteria

For the purpose of this narrative review article, the PubMed database (accessed in September 2025) has been searched with the following search terms: proteoglycan AND prostate AND cancer; *the name of each major and core matrisome proteoglycan* AND prostate AND cancer. The abbreviation and the full name of each proteoglycan were used. All of the articles obtained in this way were screened and considered, but emphasis was given to more recent articles (published within the last ten years). Articles reporting data obtained from human subjects were prioritized, but articles with only in vitro data were also considered.

## 2. Proteoglycans

Proteoglycans are ‘hybrid’ molecules consisting of a core protein and glycosaminoglycan (GAG) chains attached to it through a covalent bond. The GAG chains are made of repeated disaccharide units that are negatively charged, which makes PGs attract and hold large amounts of water within tissues. Four different types of GAG chains exist: heparan sulfate (HS), chondroitin sulfate (CS), dermatan sulfate (DS), and keratan sulfate (KS). Although PGs are classified by the type of GAG chains involved, both the protein core and the GAG chains determine the PG’s properties. Apart from their structure, the localization of PGs also plays a role in their classification. In that regard, **intracellular PGs** (serglycin) are known as well as **cell-associated PGs,** which include transmembrane syndecans and the glycosylphosphatidylinositol-anchored glypicans. Moreover, **extracellular PGs** include the hyalectans (aggrecan, versican, neurocan, and brevican) named according to their association with hyaluronan in the ECM through their N-terminus and lectins through their C-terminus. Extracellular PGs also include the small leucine-rich proteoglycans (decorin, biglycan, and lumican), and, finally, the basement membrane proteoglycans (perlecan, agrin and, collagen XVIII) [13]. More information on the form and function of PGs is available in reference [15], which provides suggestions on the nomenclature and detailed classification of proteoglycan gene families. Given the structural diversity of PGs, it is not surprising that they participate in a variety of cellular processes [16].

### Involvement of Proteoglycans in Cancer Progression

Cancer development and progression involve a multitude of intertwined processes that act either simultaneously or in isolation, with the ultimate goal of cancer growth and spread. During the last several decades of cancer research, cancer development and progression are increasingly well described and understood, and related processes are referred to under a common name of the cancer hallmarks [17,18,19]. PGs regulate and are involved in several cancer hallmarks [20]. Broken down to individual processes, a non-exhaustive list of PG’s roles in cancer includes proliferation, survival, motility, migration, invasion, morphogenesis, differentiation, cell metabolism, drug resistance, epithelial–mesenchymal transition, cell plasticity, stemness, angiogenesis, metastasis formation, etc. [20,21,22]. For example, it was shown that PGs, mainly through their GAG structural part, regulate cancer stem cell phenotypes [23] (discussed in [14]). Furthermore, PGs, again through their GAG chains and their interactions with angiogenic factors, were shown to influence vascular development [24]. Heparan sulfate proteoglycan 2 (HSPG2 or perlecan) is especially interesting in this context, since its intact protein shows pro-angiogenic properties, while one of its cleaved forms, the C-terminal fragment endorepellin, suppresses angiogenesis [25]. PGs were suggested to be involved in metastasis formation [26]; bones are among the main secondary sites for PC and it is estimated that 90% of patients with advanced PC develop secondary malignancy in the bones [27,28]. It has been known for a while that PGs are involved in bone tumor development [29].

Moreover, PGs have also been implicated in the process of autophagy, which affects resistance to therapies [30]. Finally, PGs are involved in the regulation of the immune response (discussed in [14]), an important venue to consider in attempts of cancer eradication.

These brief outlines give an overview of many of the roles of PGs in cancer progression; their specific roles in PC biology and therapy resistance are discussed in further sections.

## 3. Role of (Major) Proteoglycans in Prostate Cancer Progression

According to the classification by Alexandra Naba et al. [31,32], there are 35 core matrisome PGs (this does not include matrisome-associated PGs) in humans. However, only a small subset of them are called major PGs, and they have important biological roles that are known to a certain extent. Among **hyalectans**, versican (VCAN) and aggrecan (ACAN) are the major PGs studied in the context of PC. VCAN is a large chondroitin sulfate proteoglycan involved in cell adhesion, proliferation, migration, inflammation, angiogenesis, and tissue morphogenesis and maintenance [33,34]. On the other hand, ACAN is a component of the ECM in cartilaginous tissue [33].

Besides their localization, **small leucine-rich PGs** are classified also according to their size, as the name suggests. Among them, biglycan (BGN) is important for bone growth [35] and muscle development and regeneration. Just like other PGs from the family (decorin (DCN), fibromodulin (FMOD) and possibly lumican (LUM)), BGN also plays a role in collagen fibril assembly [33].

Perlecan (HSPG2), agrin (AGRN), and collagen XVIII belong to the **basement membrane (BM) PGs**. HSPG2 is a major component of BM and among the best studied PGs [36,37,38]. It is decorated with heparan sulfate or chondroitin sulfate GAGs. It interacts with and cross-links many ECM proteins which reflects its many roles; for example, it is involved in tissue development and in keeping the vascular homeostasis [33]. Agrin (AGRN) and collagen XVIII are other members of the ECM heparan sulfate proteoglycan family important for the development of the neuromuscular junction [33] and structural integrity of the BMs, respectively. Collagen XVIII can be cleaved to yield endostatin, a potent inhibitor of angiogenesis and, consecutively, an interesting tool in potential anti-angiogenesis treatments [39,40].

**Cell surface PGs** include syndecans, glypicans, and betaglycan (BGCAN), also known as transforming growth factor beta receptor 3, TGFβRIII. Syndecans are transmembrane heparan sulfate proteoglycans involved in cell signaling, proliferation, and adhesion. They have been shown to both promote and suppress tumor progression [41].

Glypicans are also heparan sulfate proteoglycans, but they do not span the membrane like syndecans. Instead, glypicans are attached to the cell membrane via a glycosylphosphatidylinositol anchor. They are involved in cell proliferation and motility, and morphogenesis. Their aberrant expression in some cancer types shows biomarker potential [42].

BGCAN is also a transmembrane PG involved in cell survival, stemness, differentiation, cancer metastasis, chemoresistance, and fibrosis [43]. It exerts many of its roles through TGFβ signaling; namely, BGCAN is a coreceptor for TGFβ ligands.

Finally, serglycin is the only **intracellular PG** that has been well studied. It is also the only PG decorated with heparin side chains and it is best known as a hematopoietic cell granule proteoglycan [33].

Taken together, these simple outlines suggest that the major PGs play important roles in both healthy and diseased tissues and organs. In further sections, their role in PC progression is discussed.

### 3.1. Hyalectans

The role of versican (VCAN) in prostate cancer biology is recognized in early studies [13,44] and has already been known for a while. Among the first articles on the role of VCAN in PC biology are those by David J Horsfall and colleagues. In one of their articles, the authors analyzed the expression of VCAN by immunohistochemistry in PC tissues [45]. The study showed that VCAN is expressed by periacinar and peritumoral fibromuscular stroma in benign prostatic hyperplasia and human PC tissues. Moreover, its expression was higher in the prostatic tissue of patients with early-stage PC than in non-cancer tissue. The expression of VCAN was associated with progression-free survival; namely, patients with low versican expression had significantly better prognoses. Other early studies from the same authors showed that VCAN inhibits PC cell attachment to fibronectin in vitro [46] and that VCAN was involved in PC cells crosstalk to stromal fibroblasts [47]. This gives the PC cells the ability to remodel their pericellular matrix (PCM) through recruitment of stromal VCAN, which in turn promotes their motility [47]. Besides these studies on PC cell adhesion and motility-related processes, further articles established the role of VCAN in organization of tumor spheroids, which indicated its role in cancer stem cells’ biology [48]. In accordance with this, a recent study analyzed splicing variants of versican in CD133+/CD44+ PC stem cells and showed that they are differentially expressed in PC stem cells in comparison to that of normal prostate cells [49]. Taken together, the cited articles suggest a possible role of VCAN in prostate cancer biology, especially in promotion of tumor cell motility and invasion. Additionally, there are indications that VCAN is also possibly involved in the biology of PC stem cells.

While the roles of other hyalectans such as neurocan and brevican are not thoroughly analyzed in PC, there is a study suggesting the role of aggrecan (ACAN) in formation of structures that support PC cell motility [50]. According to that article, exogenously added aggrecan stimulated thickness of the viscoelastic layer of the pericellular matrix (PCM) and promoted filopodia-like protrusions in an experiment of microrheology on cultured PC3 cells. However, the authors noted that the viscoelastic response of the PCM was not affected. While this study just scratched the surface of the possible role of ACAN in PC biology, more studies are needed to delineate its precise role.

### 3.2. Small Leucine-Rich Proteoglycans

The important role of biglycan (BGN) in PC has also been known for a while. In early gene expression studies, it was established that *BGN* is among the genes whose expression is a part of a signature that is involved in an interaction between PC and bone cells [51,52], suggesting its potential role in a metastatic milieu. In line with this, *BGN* is also among the genes (including decorin, lumican, and fibromodulin) whose expression decreases in stromal cells when grown in the presence of metastatic PC cell lines PC3 and DU145 [53]. Accordingly, a more recent gene expression study supported the potential involvement of BGN in the crosstalk between PC cells and stromal cells; namely, the study suggests that the *BGN* gene is also a part of a cancer-associated-fibroblasts (CAFs)-related prognostic model in PC [54]. Further gene expression analyses showed an overexpression of the *BGN* gene in prostate extra-capsular extension and lymph node invasion, and in clinically significant PC [55]. Moreover, its expression is part of a signature related to the worse survival of PC patients [55,56]. Finally, it is important to note that information on *BGN* gene expression is part of the commercial test Oncotype DX based on RT-PCR of 12 cancer genes and 5 reference genes that is used with prostate needle biopsies to improve treatment decisions for men diagnosed with early-stage PC [57].

Apart from the gene expression studies, other articles analyzed BGN protein expression in PC tissue. An article by Anastasia V Suhovskih and colleagues dealt with the expression of proteoglycans in normal human prostate tissue and prostate cancer by using the multiplex reverse transcription PCR and immunohistochemical analysis [58]. Eighteen clinical samples of benign prostate hyperplasia and primary PC were analyzed in total. The authors show that BGN is among the proteoglycans localized in prostate tissue stroma. In another article, BGN expression was analyzed by immunohistochemistry on a tissue microarray containing 12,427 cases of PC. The authors found that upregulation of BGN was associated with poor prognosis and PTEN deletion in PC patients [59]. An important piece of information gained from this article is that seventy-eight percent of 11,050 cancers showed BGN expression, either at low (47.7%) or high (31.1% of PCs) intensity. In accordance with this article, a more recent immunohistochemistry retrospective study on 60 cases of PC patients confirmed that BGN is expressed in cases with progression to castration-resistant PC [60].

Recently, a very important role of BGN in PC was revealed in a study that used human PC tissues and in vitro and in vivo (different mouse strains) experiments. In that study it was shown that BGN is among the three secreted proteins released by PC cells that are involved in recruitment of myeloid-derived suppressor cells (MDSCs). Therefore, this study offers valuable information regarding the efforts to restore immune surveillance in PC [61]. In line with the importance of BGN for PC growth, a recent study showed that downregulation of BGN in cancer-associated fibroblasts (CAFs) suppresses their proliferation, migration, and invasion in vitro. Moreover, in vivo xenograft assays of co-injected DU145 cells and CAFs showed that BGN expressed by CAFs positively regulates tumor growth in BALB/c nude mice [62]. Taken together, these studies suggest that BGN is a potential therapeutic target in PC.

An early article on the role of decorin (DCN) in PC [63] suggested that DCN suppresses PC growth by inhibiting cell proliferation and survival. Consequently, two articles detected lower levels of DCN in PC [64] and stromal tissue [65] compared to non-affected counterparts. Furthermore, it was shown that a recombinant oncolytic adenovirus carrying the human *DCN* gene suppressed tumor growth and skeletal metastases in a mouse model [66]. In accordance with this, a recent study showed that high DCN expression in the PC bone microenvironment indicates better prognosis after androgen deprivation therapy [67]. In summary, these studies suggest tumor- and metastasis-suppressive roles of DCN in PC. Additionally, it is worth noting that DCN is a myokine—a cytokine produced by muscle and secreted into the bloodstream—whose expression increases with exercise. There are publications that suggest the involvement of myokines and DCN specifically in exercise-induced PC suppression (reviewed in [68]).

Similarly to DCN, lumican (LUM) was also shown to suppress PC progression. Although real-time PCR and immunostaining of LUM showed its upregulation in the reactive stroma surrounding prostate tumors, in vitro and in vivo studies showed that LUM suppresses migration and the invasion of metastatic PC cells. The effect was most probably mediated through inhibition of lamellipodia and invadopodia formation [69]. In line with this, Suhovskih and colleagues reported a trend towards decreased DCN and LUM expression in PC [58]. Three further consecutive studies analyzed the biomarker potential of serum LUM in PC. The first study suggested that, among other proteins, higher plasma concentrations of LUM were associated with shorter progression-free survival in PC patients [70]. In addition to this, a more recent study that used serum samples and clinical data of 557 men who underwent radical prostatectomy for PC suggested that LUM is part of a serum biomarker quintet with a prognostic value in PC [71]. Furthermore, in the most recent article, it was suggested that LUM serum expression is part of a predictive model that is able to distinguish PC from benign prostatic hyperplasia [72].

Finally, increased expression of fibromodulin (FMOD) was reported in PC [73]. Moreover, several studies suggested its potential biomarker role in PC (for example [74,75,76]) based on the analysis of the FMOD gene and the expression of its mRNA and protein in PC tissue (reviewed in [77]). Recently, The Cancer Genome Atlas prostate adenocarcinoma mRNA expression data were analyzed using a recursive partitioning method and it was found that low expression of the *FMOD* gene is associated with worse progression-free survival of PC patients, with a Gleason score lower than 9 [78]. Taken together, these studies suggest the biomarker potential for FMOD in PC.

### 3.3. Basement Membrane Proteoglycans

Perlecan or heparan sulfate proteoglycan 2 (HSPG2) is a basement membrane protein that belongs to a group of ECM proteins that can be cleaved by proteolytic enzymes to release bioactive fragments that promote specific cellular responses. For example, it was shown that matrix metalloproteinase-7 (MMP7) cleaves HSPG2 in PC to give rise to fragments that act as potent ‘molecular switches’ which favor PC spread and invasiveness [79]. A consecutive article by the same authors based on the analyses of 288 patients who underwent radical prostatectomy revealed that HSPG2 fragments in sera and MMP-7 in tissues of PC patients are associated with PC invasiveness [80]. In continuation of these analyses, the molecular mechanisms that lead to increased invasiveness were delineated; namely, in their further research articles on this topic, Mary C. Farach-Carson and colleagues revealed that, along with released HSPG2 fragments, semaphorin 3A, focal adhesion kinase, plexin A1, and neuropilin-1 are involved in stromal invasion by PC cells [81,82]. The importance of these studies is reviewed in [83] with an interesting notion that degradation of the HSPG2-abundant stroma converts the ‘hostile’ stroma into a supporting environment that sustains cancer spread.

In another direction of research, HSPG2 and other components of the basement membrane such as diverse laminins, collagen XVIII and its cleaved endostatin-containing isoforms, were shown to be produced in an interaction between PC cells and prostate fibroblasts grown in 3D coculture spheroids [84]. It is important to note that their release is the product of a crosstalk, since separately grown PC cells and fibroblast spheroids could not recapitulate these effects. Finally, in our recent article, we studied the role of HSPG2 in prostate cancer radioresistance (more information is presented in the following sections) and we showed that high HSPG2 expression in The Cancer Genome Atlas prostate adenocarcinoma patients is correlated with worse biochemical recurrence-free survival [85], supporting the articles that suggested its potential biomarker role in PC [86].

The literature on the role of agrin (AGRN) in PC is not extensive. There is an article delineating the role of the long noncoding RNA nuclear-enriched abundant transcript 1 (NEAT1) in PC. The article showed that AGRN is a part of the signaling axis that is involved in tumor-promoting function (suppression of DNA damage, cell-cycle dysregulation, and proliferation arrest) of NEAT1 in PC cells [87].

In contrast to AGRN, the role of collagen XVIII and especially its cleaved form endostatin in PC is a bit more extensively studied. While collagen type XVIII shows both collagen and proteoglycan characteristics, endostatin itself is not a proteoglycan, but a small (20 kDa) anti-angiogenic fragment formed by a collagen XVIII cleavage. As its name suggests, endostatin is involved in the inhibition of angiogenesis, and it was shown to suppress PC growth and metastases [88,89,90,91,92].

While the studies on endostatin in PC are by far more abundant, there is a study that showed that collagen XVIII is a part of a core in vivo prostate matrisome in mass spectrometry experiments performed on tissue samples obtained from the non-cancerous areas of the surgically removed prostates [93]. This suggests its essential role in the prostate matrisome.

**Figure 1:**
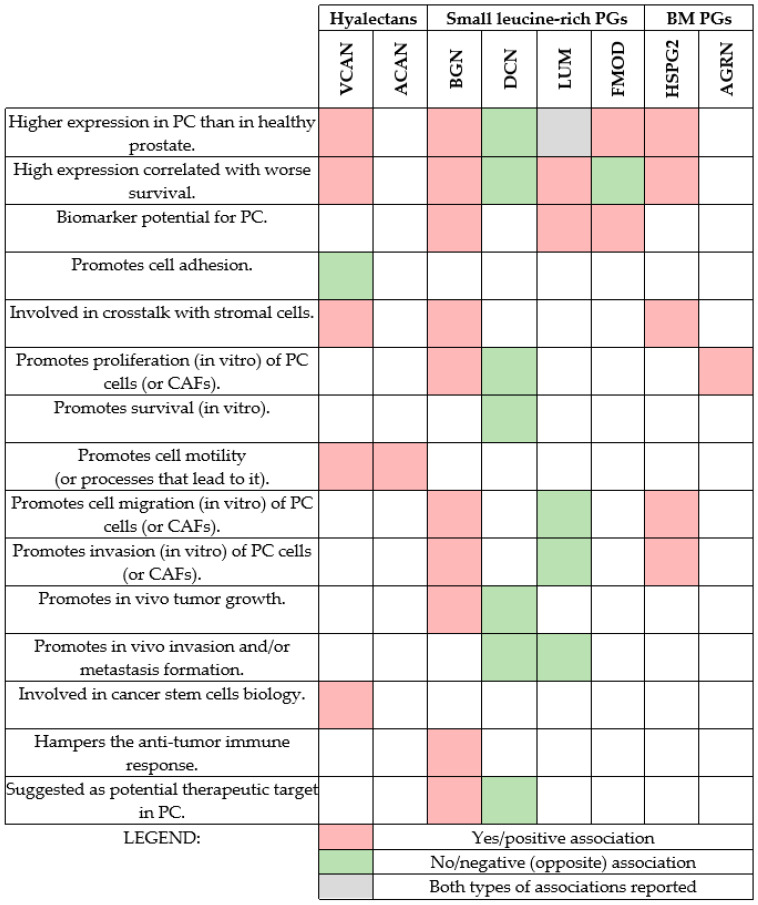
Summary of the roles of proteoglycans (PGs) in prostate cancer (PC) biology. Major PGs (hyalectans, small leucine-rich PGs, and basement membrane (BM) PGs) studied in PC are considered. Red rectangles indicate the answer is yes or has positive associations, and the green rectangles indicate negative associations or the opposite of what is written on the left side; for example, VCAN inhibits PC cell attachment to fibronectin in vitro, and DCN suppresses PC growth by inhibiting cell proliferation and survival in vitro and tumor growth and skeletal metastases in a mouse model; DCN and LUM have decreased expression in PC, etc. By looking globally, the emerging pattern suggests that DCN and LUM suppress PC progression, while all other studied PGs seem to promote it. DCN levels could potentially be increased by exercise; therefore, it is possible to enhance its levels to suppress PC.

The roles of major hyalectans, small leucine-rich proteoglycans, and basement membrane proteoglycans in PC are summarized in Figure 1.

### 3.4. Cell Surface Proteoglycans

Syndecan 1 (SDC1) is the best studied proteoglycan in PC from the syndecan family. Early studies showed that it is involved in several processes that are part of PC progression such as stabilization of tumor-initiating cells [94] and promotion of epithelial–mesenchymal transition (EMT) [95]. Moreover, its expression identifies a previously unreported cell type that is found in the stroma of PC and surrounding normal tissue but not in the healthy prostate. The authors emphasize that a subset of poor prognosis high Gleason grade PCs had a particularly high number of these cells [96]. Another article on SDC1 expression analyzed its levels in serum of PC patients and found that high levels of soluble SDC1 are an independent factor of worse PC patients’ survival [97]. The potential use of SDC1 expression as a PC biomarker was suggested also in recent studies that show that SDC1 is a part of the Appl1, Sortilin, and SDC1 biomarker panel [98,99,100,101,102,103]. In addition to this, a very recent article found that SDC1 is expressed in PC extracellular vesicles, supporting its role in PC cell communication within the body of PC patients [104]. However, it needs to be mentioned that not all articles on the role of SDC1 in PC are in unison; for example, a recent study showed that SDC1 is a part (a ligand) of a signaling axis that leads to eradication of neuroendocrine PC (NEPC) cells. NEPC is an aggressive form of PC that usually emerges as PCs become resistant to therapies [105]. This ‘dual role’ of SDC1 was noted early on; for example, Iris J. Edwards suggested in 2012 that SDC1 might have both an antagonist and agonist role in PC depending on disease stage and enzymatic conditions [13]. This was based on early articles on SDC1 expression and role in PC, all reviewed by Edwards in 2012 [13].

The articles on syndecan 2 (SDC2) in PC mainly analyzed its expression. For example, it was shown that the expression of SDC1 and SDC2 are associated with Gleason score and EMT markers in PC [106]. Moreover, SDC2 is expressed preferentially in basal cells in non-affected prostate (benign prostatic hyperplasia); however, in PC, the expression pattern shifts to granular-cytoplasmic localisation. PC patients with altered expression of SDC1 and SDC2 have worse PSA (prostate specific antigen) recurrence-free survival [107].

The expression of syndecans 3 (SDC3) and 4 (SDC4) was also studied in PC. SDC3 expression is associated with more aggressive PC tumors and a worse prognosis while SDC4 expression is associated with a better prognosis in PC patients [108]. Taken together, these studies show diverse roles and expression patterns for the members of the syndecan family in PC.

Glypican 1 (GPC1) was also suggested to be a potential biomarker in PC [109,110,111]. However, its role in PC is cell type-specific; namely, inhibition of GPC1 expression in PC-3 cells decreased their in vitro proliferation and migration while having an opposing effect in DU-145 cells. Moreover, discrepancy between the in vitro and in vivo data (GPC1 inhibition increased PC-3 tumor size in mice xenografts) was suggested to be possibly mediated by stromal cells in the tumor microenvironment [112]. Another recent article on the crosstalk with stromal cells showed that GPC1 influences the biology of human bone marrow-derived stromal cells (BSCs). More precisely, it partially regulates the phenotype of human BSCs and their transformation into activated fibroblasts, suggesting that GPC1 may be a novel target in anti-CAF therapy [113]. Other articles that dealt with GPC1 potential as a target in PC treatment [114,115,116,117] showed that Miltuximab, an antibody against GPC1, has promising safety and efficacy effects in radioimmunotherapy models of PC. This would suggest the use of GPC1 as a diagnostic molecule and a therapy target simultaneously in PC, for example, to deliver 177Lu to PC cells [116].

While glypican 2 (GPC2) was shown to promote PC proliferation, migration, and invasion [118], glypican 3 (GPC3) was suggested to be a potential target for NEPC in very recent publications [119,120]. Finally, glypican 5 (GPC5) showed a biomarker potential in PC (lower expression in PC tissue, especially in high-risk PC) [121] and inhibited PC cell proliferation and invasion. Suppression of EMT and WNT/β-catenin signaling were suggested to play a role [122]. Taken together, these articles suggest a diverse role of glypicans in PC; however, it seems that the majority of studied members of a family (excluding GPC5) have a potential to be therapeutic targets in PC.

While early studies suggested that betaglycan (BGCAN) inhibits PC growth and angiogenesis [13,123,124], more recent studies suggest its role in PC bone metastasis. First, it was shown that BGCAN is part of the signaling axis that mediates dormancy of metastatic PC in the bone. Moreover, lower BGCAN expression in PC patients correlated with increased metastatic potential and decreased survival [125], suggesting its suppressive roles in PC progression. However, a successive article suggested that BGCAN-WNT5A signaling axis stimulates PC-induced osteogenesis which contributes to PC patient morbidity and mortality [126]. This would suggest that targeting the BGCAN-WNT5A axis in PC would alleviate PC-induced osteogenesis. Further studies are needed to address these contradictory notions and to shed more light on the role of BGCAN in PC and especially its role in a bone microenvironment.

**Figure 2:**
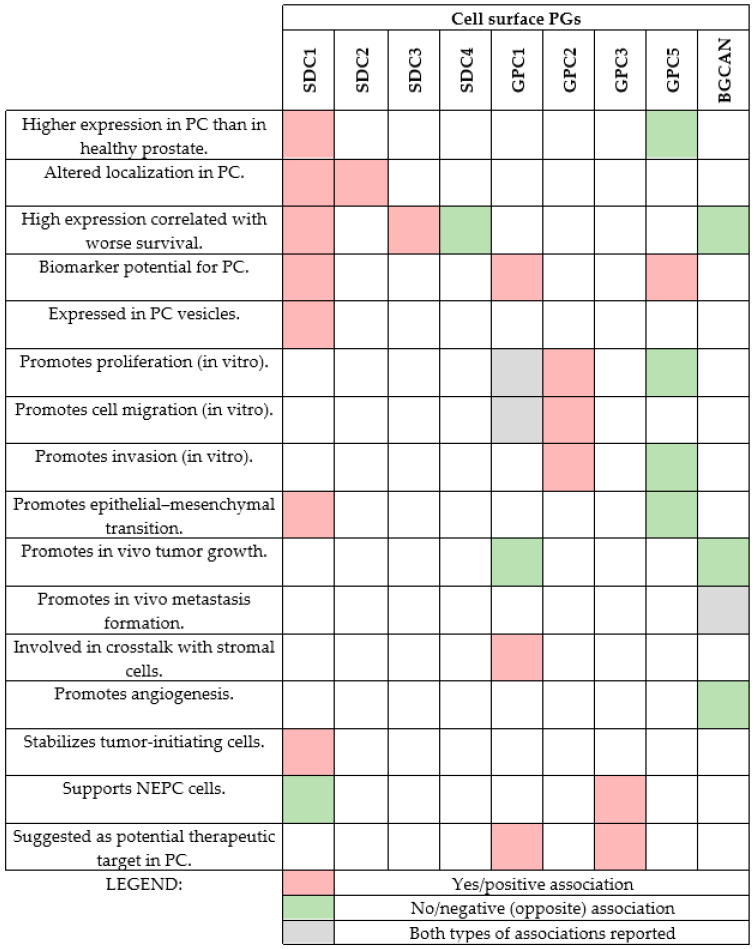
Summary of the roles of major cell surface PGs studied in PC. Red rectangles indicate the answer yes or positive associations, and the green rectangles indicate negative associations or the opposite of what is written on the left side. The gray rectangles indicate both types of association (contradicting roles reported). It is clear from the figure that SDC1 and GPC1 have been studied a little more than the rest of the proteoglycans from the family.

The roles of major cell surface proteoglycans in PC are summarized in Figure 2.

### 3.5. Intracellular Proteoglycans

Serglycin (SRGN) is among the group of proteoglycans which are under-studied in PC. However, there is an article that analyzed SRGN expression in PC and found that both the neoplastic and the normal prostatic epithelia express SRGN in cytoplasm in a granular and diffuse manner [127]. The SRGN expression was increased in high-grade adenocarcinoma in comparison to low- or moderate-grade PC cases. Additionally, the authors note that endothelial cells in tumor stroma had elevated SRGN levels [127]. While this study provides the basic information on the SRGN expression in PC, further studies are needed to dissect its precise role.

**Figure 3:**
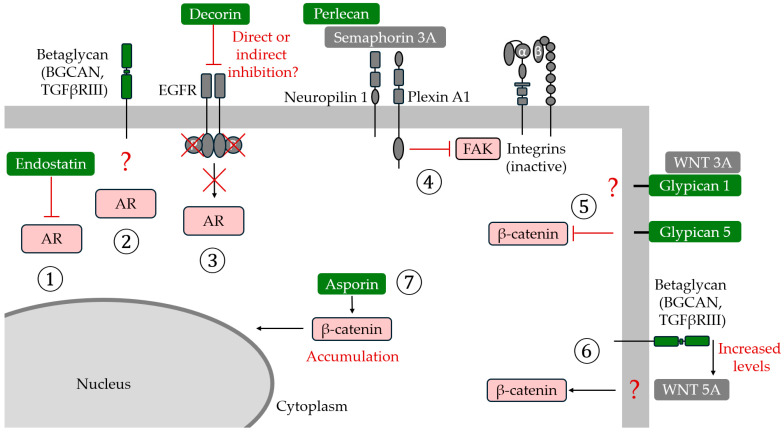
The examples of the major signaling pathways that proteoglycans interact with in prostate cancer. (1) Endostatin inhibits the androgen receptor (AR) signaling pathway, just like (3) decorin, which also inhibits the epidermal growth factor receptor (EGFR) pathway. The interaction of (2) betaglycan with AR is hypothesized. The (4) perlecan-semaphorin 3A-plexin A1-neuropilin-1 (PSPN) complex leads to focal adhesion kinase (FAK) inhibition and integrin deactivation. During PC progression, MMPs can cleave perlecan and/or Sema3A, which re-activates the integrin signaling, leading to progression to metastasis. (5) Glypican 1 was suggested to interact with WNT3A, while glypican 5 was suggested to inhibit signaling that leads to β-catenin activation. Betaglycan (6) was shown to increase WNT5A (considered to be a non-canonical WNT ligand) levels and β-catenin signaling. Finally, asporin (7) was shown to increase the expression of β-catenin and its accumulation in the nucleus.

An overview of all major and core matrisome protoglycans (according to the Edwards [13] and Naba et al. [31,32] classification) and their roles in PC are given in Table 1. Additionally, the examples of the major signaling pathways that PGs interact with in PC are depicted in Figure 3.

### 3.6. Proteoglycans and Prostate Cancer Bone Metastasis Biology

Since 90% of patients with advanced PC develop secondary malignancy in the bones [27,28], molecular processes that lead to bone metastasis formation are of utmost clinical importance. The involvement of PGs in bone tumor development has been known for a while [29], and this brief section summarizes their roles in PC bone metastasis. Several PGs have already been mentioned in that context; for example, BGN has a role in bone growth [35] and potentially promotes PC bone metastases, as revealed by its participation in gene signatures that indicate the interaction between PC and bone cells [51,52]. In contrast to BGN, DCN has been implicated in inhibition of bone metastases in a study that used mouse models [66]. Furthermore, clinical studies showed that high DCN expression in the PC bone microenvironment indicated better prognosis after androgen deprivation therapy [67]. Taken together, these studies would suggest the favorable role of DCN in suppression of PC bone metastases and the unfavorable role for BGN. However, the role of BGCAN in PC bone metastases is more complex, since two studies indicated its context-dependent roles. Namely, first it was shown that BGCAN is a part of a signaling axis that mediates dormancy of metastatic PC in bone [125], while the second study [126] found that BGCAN promotes PC-induced osteogenesis. Further studies are needed to shed more light on these complex roles. Finally, it was shown that GPC1 influences the biology of human bone marrow-derived stromal cells (BSCs) [113], confirming the involvement of PGs in different aspects of PC bone metastases biology.

## 4. Role of Proteoglycans in Prostate Cancer Therapy Resistance

Since the ultimate goal of cancer research is to find therapies that would eradicate the cancer cells or at least keep them under control, it is reasonable that the section on the role of proteoglycans in PC therapy resistance is singled out from the rest of the sections that describe their role in PC. Although not many articles have studied the role of proteoglycans in PC therapy resistance, there have been several reports in the last several years suggesting their involvement. Mechanistically, the results from these reports can be classified under several separate axes that lead to PC cell eradication: the action of docetaxel (ASPN, SDC1, and VCAN), the interference with AR signaling (BGCAN, BGN, and SPOCK1), and the effects of radiotherapy (HSPG2). For example, in a very recent article, the role of ASPN in PC and its involvement in docetaxel resistance were analyzed in in vitro and in vivo experiments [128]. Docetaxel is a taxane that binds to microtubules and subsequently triggers cell-cycle arrest and apoptosis. It is used as a part of therapy for PC patients, especially for cases with high tumor burden [149]. Additionally, there are also suggestions that docetaxel should be used in earlier stages of PC [150]. In an article on ASPN involvement, it was shown that ASPN is activated by TGFβ, and when it interacts with STMN1, it promotes PC docetaxel chemoresistance and metastasis formation. Enhanced stemness and promotion of epithelial–mesenchymal transition (EMT) were shown to be involved. Upregulation of the WNT/β-catenin signaling pathway was shown to take a part in this molecular circuitry [128]. The authors suggest that targeting the ASPN/STMN1/β-catenin axis might be a promising strategy for PC treatment.

Two earlier publications also dealt with PC resistance to docetaxel and the specific roles of SDC1 [151] and VCAN [152]. The first article showed that the levels of circulating SDC1 protein are associated with chemotherapy-resistance in 75 patients with castration-resistant PC, suggesting its biomarker role in predicting docetaxel resistance [151]. This would suggest that SDC1 levels could be possibly used to predict docetaxel resistance risk, which calls for a prospective validation. The second article investigated the role of VCAN in docetaxel-resistant PC by using in vitro models, cDNA microarray, and quantitative RT-PCR. The authors found that targeting VCAN is a potential therapeutic strategy in docetaxel-resistant PC [152].

In a more recent article, the expression of BGCAN was studied in patients with metastatic castration-resistant PC, as part of the PROMOTE study. The authors analyzed the resistance mechanisms to abiraterone acetate/prednisone (AA/P) therapy. AA/P is a combination therapy for advanced PC, in which abiraterone acetate blocks androgen production, and prednisone, a steroid, is added to prevent side effects from therapy. In a cited article, by using the RNA seq-technology on biopsy tissues from 83 patients before and after AA/P treatment, the authors found that nonresponders to therapy had low expression of BGCAN, suggesting BGCAN as a most prominent ‘nonresponder biomarker‘ [153]. Although the mechanisms of action of BGCAN have not been delineated, given the frequent role of TGFβ signaling in crosstalk with different signaling pathways, it could be possible that BGCAN might be involved in a crosstalk with androgen receptor and intersect with androgen receptor plasticity [154]. Regardless of the mechanism, a window-of-opportunity biopsy study would be helpful to further analyze the suitability of BGCAN as a ‘nonresponder biomarker‘.

Another recent article analyzed the composition of proteomes from the biopsies of PC patients that rapidly progress from hormone-sensitive PC to more severe castration-resistant PC, as a part of a retrospective study [155]. The authors found that BGN is one of the two proteins associated with a fast progression from hormone-sensitive to castration-resistant PC.

Another recent article investigated the role of extrachromosomal circular DNA (eccDNA)-related gene *SPOCK1* in PC enzalutamide resistance [140]. Enzalutamide is a hormone therapy drug (an androgen receptor inhibitor) used to treat different types of PC. The authors showed that the *SPOCK1*-associated eccDNA contributes to PC enzalutamide resistance and that the mechanism of regulation of EMT is involved.

Finally, our group has recently studied the composition of integrin adhesion complexes of DU145 radiation sensitive (parental) and radioresistant cell lines by using proteomics [85]. Slightly less than half of patients with localized PC are treated with radiotherapy. However, depending on the clinical stage, it is estimated that 20–40% of PC patients will develop recurrence after treatment. Therefore, PC radioresistance is an obstacle for successful PC treatment. In our article, we found that the radioresistant DU145 cell line re-organizes the composition of its ECM proteins. Among the many ECM proteins that change expression in DU145 radioresistant cell line, we singled out HSPG2 protein; its downregulation sensitizes DU145 radioresistant cells to irradiation while leaving the sensitivity of DU145 parental cells unchanged. This suggests that HSPG2 might be a potential therapeutic target in PC. Further studies on the analysis of HSPG2 protein expression combined with radiotherapy to build up the radioresistance score are necessary.

In conclusion, diverse proteoglycans seem to be involved in PC resistance to major lines of therapies that are used to eradicate PC. Their potential roles as therapeutic targets in different diseases are reviewed in [156]. Table 2 lists the related publications discussing the role of proteoglycans in PC therapy resistance.

## 5. Conclusions

PGs are a major constituent of the ECM, and as such, an important part of the TME. Given their structural diversity and the plentitude of interacting proteins, as well as their strategic location within the TME, it is not surprising that these molecules play important roles during cancer progression. Although the therapeutic potential of PGs is, in general, considered underexploited [156], there is a considerable body of evidence in the literature suggesting that this class of molecules could be used as both biomarkers and therapy targets (Table 3) in different cancer types. In this review article, several PGs emerged as potential biomarkers and/or therapy targets in PC. However, further studies are needed in order to bridge the gap between these initial suggestions and their use in clinics.

## Figures and Tables

**Table 1:** Role of major and core matrisome proteoglycans (according to the Edwards [13] and Naba et al. [31,32] classification) in prostate cancer progression and their possible biomarker potential. The potential of proteoglycans as PC biomarkers is just briefly outlined; a more in-depth overview is presented in [77].

Proteoglycan	Reported Findings	Year	Reference
Aggrecan (ACAN)	Stimulates motility-related processes in an experiment of microrheology on cultured PC3 cells.	2012	[50]
Agrin (AGRN)	CDC5L-AGRN signaling mediates the PC-promoting function of the long noncoding RNA NEAT1.	2018	[87]
Asporin (ASPN)	Promotes PC metastasis through the Wnt/β-catenin signaling pathway; enhanced stemness and epithelial–mesenchymal transition (EMT) are involved.	2025	[128]
	Promotes PC metastatic progression; restriction of mesenchymal stromal cell differentiation and alteration of the tumor microenvironment are involved.	2019	[129]
	In a cohort of 326 PC patients, increased expression of ASPN was correlated with decreased time to biochemical recurrence.	2017	[130]
Betaglycan (BGCAN)	Drives PC-induced osteogenesis.	2019	[126]
	Part of the signaling axis that mediates dormancy of metastatic PC in the bone.	2018	[125]
	Inhibits PC growth and angiogenesis.	2012, 2007, 2005	[13,123,124]
Biglycan (BGN)	Downregulation of BGN in cancer-associated fibroblasts suppresses cell proliferation, migration, and invasion in vitro, and in vivo xenograft assays.	2025	[62]
	Among the key secretome factors that regulate recruitment of myeloid-derived suppressor cells in PC.	2023	[61]
	Upregulation associated with poor prognosis and PTEN deletion in PC patients.	2023, 2017	[59,60]
	Potentially promotes PC bone metastasis.	2006	[51]
Decorin (DCN)	High DCN expression in the PC bone microenvironment indicates better prognosis after androgen deprivation therapy.	2025	[67]
	Lower expression in PC than benign prostatic hyperplasia tissue has a potential prognostic value.	2020	[64]
	Inhibits bone metastasis.	2015	[66]
	Reduced expression in PC stroma compared to non-malignant prostate stroma.	2012	[65]
	Suppresses PC growth.	2009	[63]
Endocan (ESM1)	Interaction of β-catenin with nuclear ESM1 promotes stemness of metastatic PC.	2021	[131]
	Tissue endocan expression level is higher in PC patients compared to those with benign prostate hyperplasia.	2021	[132]
	Overexpression of ESM1 in PC correlates with Gleason score and androgen receptor expression.	2017	[133]
	ESM1 downregulation decreases migration in PC cells.	2017	[134]
	Loss of ESM1 expression promotes PC tumorigenicity and metastasis.	2017	[135]
	Associated with tumor recurrence in PC.	2017	[136]
Fibromodulin (FMOD)	Potential biomarker in PC.	2024, 2023	[76,77,78]
Glypican 1 (GPC1)	PC target.	2021, 2020, 2016	[114,115,116,117]
	Expressed in PC cell lines; interacts with WNT3A.	2021	[137]
	Influences the biology of human bone marrow-derived stromal cells and PC cell aggressiveness.	2021	[113]
	Part of a MiCheck test for aggressive PC.	2020	[111]
	The role of GPC-1 in PC is cell type-specific; discrepancy between the in vitro and in vivo data possibly mediated by stromal cells in the tumor microenvironment.	2019	[112]
	PC biomarker.	2018, 2018	[109,110]
Glypican 2 (GPC2)	Promotes PC proliferation, migration, and invasion.	2024	[118]
Glypican 3 (GPC3)	Potential target for neuroendocrine PC.	2025, 2023	[119,120]
Glypican 5 (GPC5)	Inhibits PC cell proliferation and invasion; suppression of EMT and WNT/β-catenin signaling are involved.	2018	[122]
	Potential diagnostic and prognostic PC biomarker (lower expression in PC tissue, especially in high-risk PC).	2016	[121]
Lumican (LUM)	Part of a serum biomarker signature to (a) distinguish PC from benign prostatic hyperplasia and (b) predict biochemical recurrence and adverse pathology.	2023, 2021, 2020	[70,71,72]
	LUM in the reactive stroma has a suppressive role on the PC progression.	2013	[53,69]
Osteoglycin (OGN)	PC patients with high OGN expression show better survival.	2024	[138]
Perlecan (HSPG2)	High expression correlates with worse survival of The Cancer Genome Atlas prostate adenocarcinoma patients.	2024	[85]
	HSPG2 cleavage triggers PC cell dyscohesion, migration, and tissue invasion.	2021, 2018, 2016, 2014	[79,80,81,82]
	HSPG2 expression in PC tissues correlates with a high Gleason score and rapid cell proliferation; inhibition of HSPG2 expression in PC cell lines decreases cell growth and Sonic Hedgehog signaling.	2006	[139]
Serglycin (SRGN)	Detected in both the neoplastic and the normal prostatic epithelia.	2015	[127]
Syndecan 1 (SDC1)	Found in PC extracellular vesicles.	2025	[104]
	Potential PC biomarker (part of the Appl1, Sortilin, and SDC1 biomarker panel).	2024, 2023	[98,99,100,101,102,103]
	Part of the signaling axis that promotes the release of TNFα by mast cells to suppress neuroendocrine PC.	2024	[105]
	SDC1 expression identifies a previously unreported cell type that is frequent in a subset of poor prognosis high Gleason grade tumors.	2017	[96]
	Soluble SDC1 serum level is an independent pre-operative predictor of cancer-specific survival in PC.	2016	[97]
	Mediates EMT in PC.	2016	[95]
	Contributes to PC progression by stabilizing tumor-initiating cells.	2013	[94]
Syndecan 2 (SDC2)	SDC2 is expressed preferentially in basal cells in non-affected prostate; in PC the expression pattern shifts to granular-cytoplasmic localization, and PC patients with altered expression have worse PSA recurrence-free survival.	2011	[107]
	The expression of SDC2 is associated with Gleason score and EMT markers in PC.	2010	[106]
Syndecan 3 (SDC3)	SDC3 expression is associated with more aggressive PC tumors and a worse prognosis.	2021	[108]
Syndecan 4 (SDC4)	SDC4 expression is associated with a better prognosis in PC patients.	2021	[108]
Testican 1 (SPOCK1)	Extrachromosomal circular DNA-related SPOCK1 contributes to the development of PC; regulation of epithelial–mesenchymal transition (EMT) is involved.	2024	[140]
	High SPOCK1 expression is associated with advanced PC.	2019	[141]
	SPOCK1-snail/slug axis is involved in EMT; its targeting contributes to inhibition of PC metastasis.	2019	[142]
	Promotes tumor growth and metastasis in human PC.	2016	[143]
	Upregulation of SPOCK1 mRNA and protein in PC samples.	2015	[144]
Testican 2 (SPOCK2)	Upregulation of SPOCK2 inhibits the invasion and migration of PC cells; MT1-MMP/MMP2 pathway is involved.	2019	[145]
Testican 3 (SPOCK3)	SPOCK3 expression is associated with immune cell infiltration; PC patients with higher SPOCK3 expression show better disease-free survival.	2023	[146]
	Lower expression in bone metastasis than in primary PC.	2022	[147]
	Among the genes with the most downregulated expression in PC lymph node and liver metastases compared to primary tumors.	2021	[148]
Versican (VCAN	Promotes PC cell motility and invasion.	2012, 2007	[13,47]

**Table 2:** Role of proteoglycans (according to the Edwards [13] and Naba et al. [31,32] classification) in prostate cancer therapy resistance.

Proteoglycan	Reported Findings	Year	Ref.
Asporin (ASPN)	Promotes PC docetaxel chemoresistance through the Wnt/β-catenin signaling pathway.	2025	[128]
Betaglycan (BGCAN)	Among other characteristics, patients with castration-resistant PC non-responding to abiraterone/prednisone treatment had low expression of BGCAN.	2022	[153]
Biglycan (BGN)	One of the two proteins associated with a fast progression from hormone-sensitive to castration-resistant PC.	2023	[155]
Perlecan (HSPG2)	Regulates radioresistance in PC DU145 cells.	2024	[85]
Syndecan 1 (SDC1)	Circulating SDC1 is associated with chemotherapy-resistance in castration-resistant PC.	2018	[151]
Testican 1 (SPOCK1)	Extrachromosomal circular DNA-related gene SPOCK1 contributes to PC enzalutamide resistance; regulation of EMT is involved.	2024	[140]
Versican (VCAN)	Potential therapeutic target in docetaxel-resistant PC.	2015	[152]

**Table 3:** Examples of emerging treatment strategies targeting proteoglycans.

Strategy	Example	Comment
Antibodies against PGs	Antibodies against different glypicans	Targeting cell surface PGs.
Interference with enzymes that process PGs	Heparanase inhibitors	Block the activity of enzymes that modify, e.g., heparan sulfate chains.
Modification of PGs	Enzymatic glycosaminoglycan (GAG) editing	Methods include synthesis of new GAG chains, degradation of existing GAGs or production of GAGs with specific characteristics.

## Data Availability

No new data were created or analyzed in this study.

## Abbreviations

The following abbreviations are used in this manuscript:

ECM	Extracellular matrix
TME	Tumor microenvironment
PGs	Proteoglycans
PC	Prostate cancer
GAG	Glycosaminoglycan
HS	Heparan sulfate
CS	Chondroitin sulfate
DS	Dermatan sulfate
KS	Keratan sulfate
HSPG2	Heparan sulfate proteoglycan 2
VCAN	Versican
ACAN	Aggrecan
BGN	Biglycan
DCN	Decorin
FMOD	Fibromodulin
LUM	Lumican
AGRN	Agrin
BM	Basement membrane
BGCAN	Betaglycan (also known as transforming growth factor beta receptor 3, TGFβRIII)
PCM	Pericellular matrix
CAFs	Cancer-associated-fibroblasts
PTEN	Phosphatase and tensin homolog
MDSCs	Myeloid-derived suppressor cells
MMP7	Matrix metalloproteinase-7
NEAT1	Nuclear-enriched abundant transcript 1
SDC1	Syndecan 1
EMT	Epithelial–mesenchymal transition
NEPC	Neuroendocrine prostate cancer
PSA	Prostate specific antigen
GPC1	Glypican 1
BSCs	Bone marrow-derived stromal cells
WNT	Wingless-related integration site
SRGN	Serglycin
AA/P	Abiraterone acetate/prednisone therapy
eccDNA	Extrachromosomal circular DNA
